# Adolescent kratom exposure affects cognitive behaviours and brain metabolite profiles in Sprague-Dawley rats

**DOI:** 10.3389/fphar.2022.1057423

**Published:** 2022-11-28

**Authors:** Aiman Nadhirah Zul Aznal, Nurul Aqmar Mohamad Nor Hazalin, Zurina Hassan, Noorul Hamizah Mat, Nelson Jeng-Yeou Chear, Lay Kek Teh, Mohd Zaki Salleh, Farah Wahida Suhaimi

**Affiliations:** ^1^ Centre for Drug Research, Universiti Sains Malaysia, Pulau Pinang, Malaysia; ^2^ Integrative Pharmacogenomics Institute (iPROMISE), Universiti Teknologi MARA, Cawangan Selangor, Kampus Puncak Alam, Puncak Alam, Malaysia

**Keywords:** *M. speciosa*, kratom, mitragynine, adolescence, substance abuse, cognition, metabolomic

## Abstract

Adolescence is a critical developmental period during which exposure to psychoactive substances like kratom (*Mitragyna speciosa*) can cause long-lasting deleterious effects. Here, we evaluated the effects of mitragynine, the main alkaloid of kratom, and lyophilised kratom decoction (LKD) on cognitive behaviours and brain metabolite profiles in adolescent rats. Male Sprague-Dawley rats (Postnatal day, PND31) were given vehicle, morphine (5 mg/kg), mitragynine (3, 10, or 30 mg/kg), or LKD (equivalent dose of 30 mg/kg mitragynine) for 15 consecutive days. Later, a battery of behavioural testing was conducted, brain was extracted and metabolomic analysis was performed using LCMS-QTOF. The results showed that mitragynine did not affect the recognition memory in the novel object recognition task. In the social interaction task, morphine, mitragynine, and LKD caused a marked deficit in social behaviour, while in Morris water maze task, mitragynine and LKD only affected reference memory. Metabolomic analysis revealed distinct metabolite profiles of animals with different treatments. Several pathways that may be involved in the effects of kratom exposure include arachidonic acid, pantothenate and CoA, and tryptophan pathways, with several potential biomarkers identified**.** These findings suggest that adolescent kratom exposure can cause cognitive behavioural deficits that may be associated with changes in the brain metabolite profiles.

## 1 Introduction

The emergence of kratom (*Mitragyna speciosa* Korth.) as a “new” substance that is cheaper and more easily accessible has enticed people to seek it as an alternative to other banned narcotics (Suhaimi et al., 2016; Leong Bin Abdullah and Singh, 2021). However, the trend of recreational use of kratom has spread to a younger population, causing an alarming concern in society (Halim et al., 2021). Regular kratom consumption during adolescence is of particular concern as adolescents are more vulnerable to early substance abuse (Spear, 2016; Gorey et al., 2019; Salmanzadeh et al., 2021). Adolescents’ brains are still developing and malleable, and thus the drug-induced developmental changes in neurochemical processes will further increase the risk of lifelong substance use disorder.

Multiple studies have shown that chronic substance abuse such as morphine, methamphetamine, cannabis, and cigarette smoking can lead to cognitive behavioural deficits (Sofuoglu et al., 2013; Han et al., 2014; Thomas-Roig et al., 2016; Moazen et al., 2018; Gorey et al., 2019; Verdejo-Garcia et al., 2022). The severity of the cognitive deficits depends on the age of onset of substance abuse with more significant impairment associated with early use (Jordan and Andersen, 2017). Since kratom has been claimed to have both therapeutic and addictive potentials (Gutridge et al., 2020; Hassan et al., 2020; Vicknasingam et al., 2020; You et al., 2022), the widespread use of kratom in adolescents further highlighted the need for a better understanding of the effects of adolescent kratom exposure on multiple aspects including cognitive behaviours.

Despite the growing interest in the pharmacological profile of kratom, their precise neurochemical impacts remain unclear. However, using metabolomics, kratom-induced changes in neurochemical processes, especially in the brain may be elucidated (Caspani et al., 2021). The diversity of the metabolites which are the end-products and by-products of complicated biosynthetic and catabolism pathways is of great significance to the metabolome (Zaitsu et al., 2013). Moreover, several metabolomic studies on other drugs of abuse such as methamphetamine have demonstrated that the metabolic alterations were associated with neuronal and energy metabolism disruptions in various biological samples (Bustamante et al., 2002; Adkins et al., 2013; Zheng et al., 2014; Kim et al., 2019). Meanwhile, investigation on morphine or heroin exposure revealed various metabolic pertubations that was dependent on the dose and length of the drug administration, and these include the amino acid metabolism, energy metabolism and neurotransmitters (Hu et al., 2012; Li et al., 2017). These findings further indicate that a comprehensive analysis of the metabolome will be highly useful in detecting the metabolic disturbances that may be related to the adverse effects and discovering any potential kratom addiction biomarkers or therapeutic targets (Adkins et al., 2013; Kim et al., 2019).

Therefore, the present study aims to elucidate the effects of mitragynine, the main alkaloid of kratom, and lyophilised kratom decoction during adolescence on cognitive and behavioural performances using a battery of behavioural testing. We further asked if adolescent exposure to mitragynine and lyophilised kratom decoction could alter the brain metabolic profiles using an LCMS-QTOF-based metabolomic study.

## 2 Materials and methods

### 2.1 Animals

Subjects were adolescent (postnatal days 21–30, PND21-30, ∼80 g, Hunt et al., 2016) male Sprague-Dawley rats obtained from Advanced Medical and Dental Institute, Universiti Sains Malaysia (USM). Animals were kept in a conventional laboratory setting with a temperature set at 23°C ± 2°C and a 12 h/12 h light-dark cycle (lights on at 0700). Animals were fed an altromin food diet and had free access to water. Prior to the trial, the animals were acclimatised to the facility for at least 1 week and handled for 5 min/day during this adaptation period. All protocols were approved by the USM Institutional Animal Care and Use Committee with no: USM/IACUC/2019/(120)(1022).

### 2.2 Drugs preparation

Morphine hydrochloride was purchased from Labchem (Lipomed). Morphine has been widely used as a comparison for mitragynine for cognitive assessment (You et al., 2022; Yusoff et al., 2016) and other behavioural studies (Harun et al., 2015). Mitragynine was isolated from kratom (*M. speciosa*) leaves according to our in-house method (Chear et al., 2021) with a purity of approximately 98%. Mitragynine was kept at −20°C until further use. The doses of mitragynine (3, 10, or 30 mg/kg) were selected based on previous studies (Harun et al., 2015; Yusoff et al., 2016). Lyophilised kratom decoction (LKD) was prepared in-house (Supplementary Material S1). Mitragynine was freshly prepared daily in 20% Tween-20 while morphine and LKD were prepared in distilled water.

### 2.3 Experimental design

Animals were divided into six groups. Group 1 was given vehicle orally (p.o.). Group 2 was given morphine (5 mg/kg; i.p., Yusoff et al., 2016), and groups 3, 4, and 5 were given different doses of mitragynine (3, 10, or 30 mg/kg, p.o.), respectively. Group 6 was assigned LKD which dose equivalent to 30 mg/kg of mitragynine (p.o., Yusoff et al., 2016). The groups are as follows:

Group 1: Vehicle (p.o.; Veh)
Group 2: Morphine (5 mg/kg, i.p.; Mor)
Group 3: Mitragynine (3 mg/kg, p.o.; Mit 3)
Group 4: Mitragynine (10 mg/kg, p.o.; Mit 10)
Group 5: Mitragynine (30 mg/kg, p.o.; Mit 30)
Group 6: LKD (p.o.)

**FIGURE 1:**
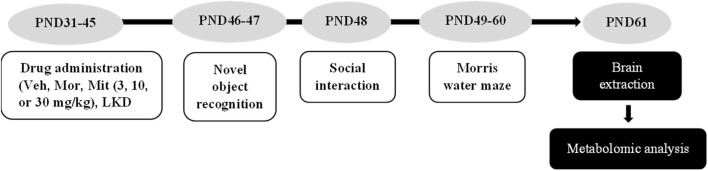
Drug administration, behavioural testing, and metabolomic analysis timeline.

Animals were administered with respective drugs for 15 consecutive days. Twenty-4 h after the last drug’s administration, a battery of behavioural testing was conducted. At the end of behavioural testing, animals treated with vehicle, morphine, low and high doses of mitragynine (3 and 30 mg/kg), and LKD were euthanized with sodium pentobarbital (100 mg/kg, i.p.). The brain was quickly removed and snap-frozen using liquid nitrogen to minimize metabolite degradation and kept at −80°C until further use. The timeline is depicted in Figure 1.

### 2.4 Behavioural testing

#### 2.4.1 Novel object recognition

The novel object recognition test (NORT) evaluates object recognition memory which takes advantage of the rat’s innate tendency to explore novel object more than the familiar object. The protocols were conducted in accordance with Bevins and Besheer (2006), with slight modifications. Initially, animals were habituated to the empty black box (45 cm × 45 cm × 45 cm) for 5 min. The next day, the animals were familiarized with two identical objects; objects A1 and A2. The animals were allowed to explore and interact with both objects for 5 min and then returned to their home cage. After 1-h retention interval, the first recognition phase was performed. In this phase, the familiar object A2 was replaced with a novel object (B1), but the objects’ positions remained unchanged. The animals were allowed to explore the objects for 5 min. To assess long-term recognition memory, the animals were introduced to a new object (C1) after 24-h retention interval, in the presence of a familiar B1 object. The arena was thoroughly cleaned between sessions with 70% alcohol to remove the odour of the previous animal. The experiment was video recorded, and the time spent on each object as well as the number of direct contacts were analysed. Direct contact was defined as the nose coming into contact with or being directed at the object within a minimum distance (≤1 cm). A discrimination ratio greater than 0.5 indicates a preference for and increased interaction with the novel object (Bevins and Besheer, 2006; You et al., 2022). The discrimination ratio was calculated as below:

Discrimination ratio = time spent on the novel object (s)/total time spent on both objects (s).

#### 2.4.2 Social interaction test

The social interaction (SI) test was performed in an open black square arena (80 cm × 80 cm × 30 cm). A pair of animals with a treatment-matched unfamiliar conspecific of similar body weight was placed at the opposite corners in the arena. The animals were allowed to freely roam the arena for 10 min and then returned to their home cage. The amount of time the animals spent engaging with one another was video recorded. Scored behaviours include active (i.e., sniffing, following, and grooming a conspecific) and passive behaviours (i.e., animals lying next to each other; Almeida et al., 2013). The entire apparatus was wiped and sanitised with 70 percent ethanol before each session to reduce the strong interaction between them.

#### 2.4.3 Morris water maze

The Morris water maze (MWM) task was used to assess hippocampal-dependent spatial learning in which subjects navigate from starting positions along the perimeter of the water maze’s wall to a hidden escape platform using distal cues. MWM was conducted as described previously (Ku et al., 2016; Hassan et al., 2019). A circular black pool (diameter, 160 cm) was filled with opaque water (25°C ± 1°C) to a depth of 50 cm. The maze was divided into four virtual quadrants, with an escape platform (diameter, 10 cm) submerged 2 cm below the water surface at the centre of one of the quadrants. On the habituation day, the animal was gently released into the pool and allowed to swim for 60 s without the escape platform. On the following day, animals received four acquisition trials per day over five consecutive days, with the order of starting positions randomized. The trial was called off if the animal escaped onto the platform or if 60 s had elapsed. The animal was allowed to stay on the escape platform for 30 s once it reached the platform. Animals that did not find the escape platform was guided by the experimenter and allowed to stay on the platform for 30 s. After 24 h of the last acquisition trial, a probe trial was performed. The hidden platform was removed, and animals were allowed to swim for 60 s. The amount of time spent in the target quadrant was recorded as a measure of spatial reference memory. For reversal training, each animal was given four trials per day for 5 days as in initial acquisition trials, but with a reversed location of the hidden platform. A reversal probe trial was conducted 24 h later. Finally, a cued learning trial was conducted in the presence of a visible escape platform to assess general sensorimotor performance (Ku et al., 2016; Damodaran et al., 2018).

### 2.5 Metabolomic analysis

The samples were analysed using LCMS-QTOF (model 6520 Agilent Technologies, SA, United States) with a ZORBAX Eclipse plus C18 column (100 mm × 2.1 mm × 1.8 μm, Agilent Technologies, SA, United States) as described previously (Rosdy et al., 2021). The system was operated at a flow rate of 0.25 ml/min with solvent A (water with 0.1% formic acid) and solvent B (acetonitrile with 0.1% formic acid), over a gradient of 0–36 min with an increasing percentage of B from 5% to 95%. The mass spectrometry was operated in electrospray ionization (ESI) positive mode. To ensure mass accuracy, two reference masses of 121.0505 m/z (C5H4N4) and 922.0098 m/z (C18H18O6N3P3F24) were used.

Initially, the brain samples were weighed, homogenized, and bathed with 300 μl chloroform, 300 μl methanol, and 300 μl water (for every 30 mg of the brain). Then, samples were vortexed for 60 s and centrifuged for 10 min at 5°C (10,000 rpm or 9,408 × g; Centrifuge PK 121R, ALC, United Kingdom). The upper phase (methanol and water) was separated from the lower phase (chloroform). Acetonitrile was added with a ratio of 1 acetonitrile: 2 methanol-water volume and centrifuged for 10 min (10,000 rpm or 9,408 × g, 5°C). The supernatants were collected into new centrifuge tubes and were vacuum dried using a centrifuge concentrator (Concentrator plus, Eppendorf, United States). All vacuum dried samples were reconstituted with 40 μl of water and acetonitrile with a ratio of 1:1 and vortexed again for 60 s before centrifugation (10,000 rpm or 9,408 × g, 10 min, 5°C). The supernatant (30 µl) was transferred into an insert and then transferred into an injection vial (Rosdy et al., 2021).

The samples were run in 14 batches, each consisting of six samples, one blank and one quality control (QC). The analysis was performed in four technical replicates. QC samples were prepared from the aliquot of 14 batches of pooled samples. They were injected independently at the beginning, middle, and end of the run to check the system stability and performance, and the consistency of sample preparation for each batch of analysis. QC samples were evaluated by analysing the relative standard deviation (RSD) of selected metabolites that are constantly present in 80% of the pooled samples.

### 2.6 Data analysis

For the behavioural study, all data were expressed as mean ± standard error of the mean (SEM) using GraphPad Prism version 6.00 for Windows, GraphPad Software, La Jolla California United States. Data were analysed using the one-way, two-way or repeated-measures ANOVA followed by the Bonferroni or Tukey’s *post-hoc* test when the data showed normality. If the data did not exhibit normal distribution, a Kruskal-Wallis ANOVA and Dunnet *post-hoc* test was employed. The *p-*value < 0.05 was considered statistically significant.

For the metabolomic study, each sample was analysed in four technical replicates. The raw data was collected over the full scan mode from 100 to 1,000 m/z. Metabolite raw data were processed using Agilent Mass Hunter Qualitative Analysis B.05.00 software (Agilent Technologies, Santa Clara, CA, United States) to remove background noise and normalize the data. For statistical analysis, Agilent Mass Hunter Mass Profiler Professional (MPP) software version B.12.01 (Agilent Technologies, Santa Clara, CA, United States) was used for data filtering, statistical analysis, and metabolites identification. The analysis included are two-way ANOVA, fold chain, and principal component analysis (PCA). The metabolites detected were determined by ID Browser Identification in Agilent Mass Hunter Mass Profiler Professional (MPP) software. Recursive analysis was subsequently performed to remove false-positive data. The identified compounds were annotated using METLIN (http://metlin.scripps.edu/) and KEGG (http://www.genome.jp/kegg/) databases. Pathway analysis of the metabolites was analysed using Metaboanalyst software.

## 3 Results

### 3.1 Effects of early adolescent kratom exposure on recognition memory in NOR task

**FIGURE 2:**
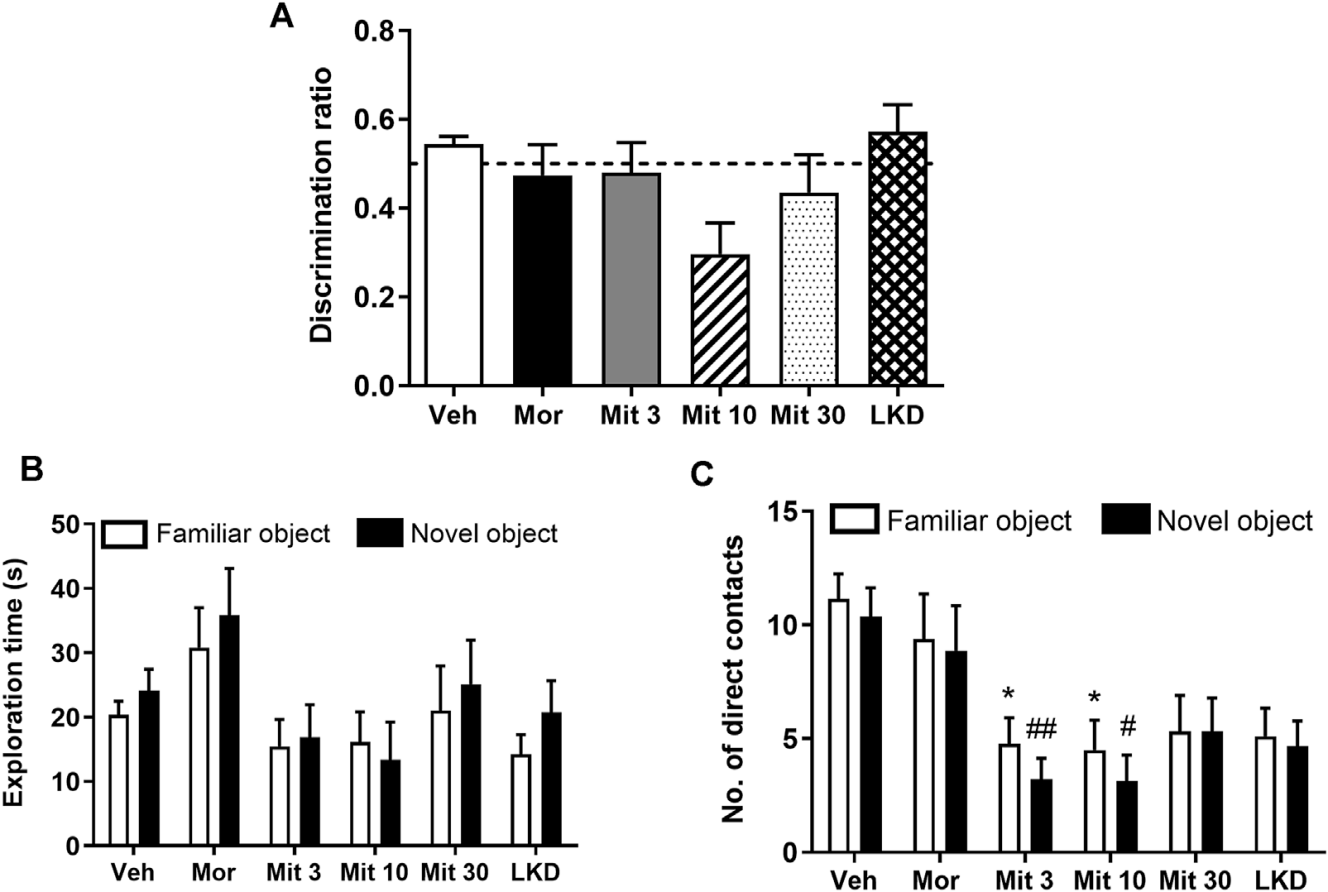
Effects of adolescent kratom exposure on **(A)** Discrimination ratio after 1-h retention interval **(B)** Exploration time on both familiar and novel objects, and **(C)** Number of direct contacts with both familiar and novel objects. Data are expressed as mean ± SEM (*n* = 8–10). *p < 0.05, vs. familiar object of the vehicle-treated group. ^#^ *p* < 0.05, ^##^ *p* < 0.01, vs. novel object of the vehicle-treated group.

One-way ANOVA showed no significant effect of treatment on discrimination ratio (*F*_5, 47_ = 2.129, *p* = 0.0783) in a novel object recognition task after a 1-h retention interval (Figure 2A). Two-way ANOVA showed a significant effect of treatment (*F*_5, 98_ = 3.251, *p* = 0.0093) but not the objects (*F*_1, 98_ = 1.024, *p* = 0.3140) on the exploration time (Figure 2B). However, no effect of interaction was observed (*F*_5, 98_ = 0.1964, *p* = 0.9632). The number of direct contacts was also affected by the treatment (*F*_5, 98_ = 10.16, *p* < 0.0001, Figure 2C) but no significant effects of the object (*F*_1, 98_ = 0.9418, *p* = 0.3342) and interaction was found (*F*_5, 98_ = 0.08853, *p* = 0.9939). *Post-hoc* test analysis further showed that mitragynine significantly reduced the number of direct contacts with the familiar object (Mit 3, *p* = 0.0415; Mit 10, *p* = 0.0381) and the novel object (Mit 3, *p* = 0.0091; Mit 10, *p* = 0.0124).

**FIGURE 3:**
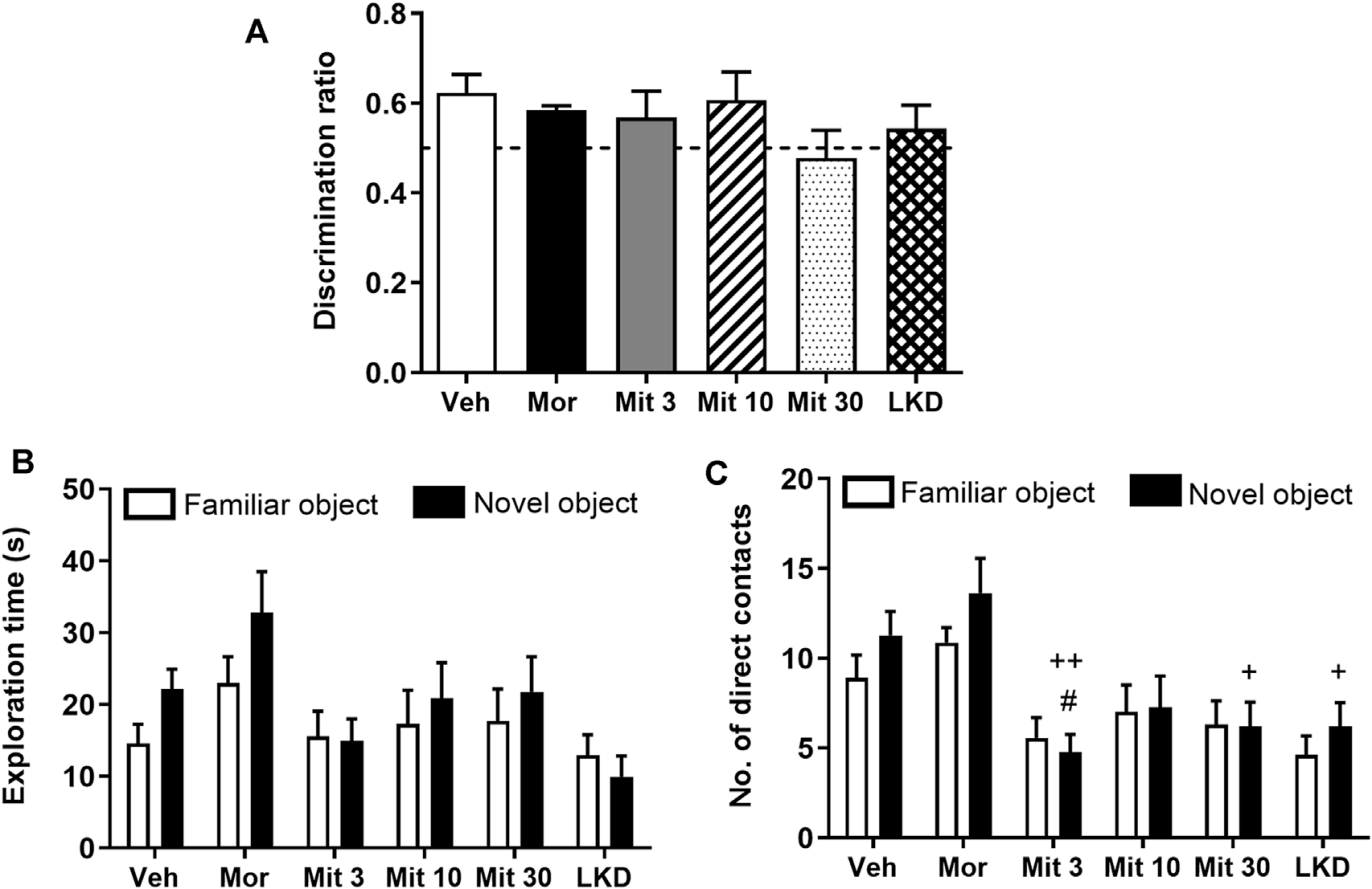
Effects of adolescent kratom exposure on **(A)** Discrimination ratio after 24-h retention interval, **(B)** Exploration time on both familiar and novel objects, and **(C)** Number of direct contacts with both familiar and novel objects. Data are expressed as mean ± SEM (*n* = 8–10). ^#^ *p* < 0.05, vs. novel object of the vehicle-treated group; ^+^ *p* < 0.05, ^++^ *p* < 0.01, vs. novel object of the morphine-treated group.

When the retention interval was prolonged to 24 h in the second testing phase, no significant difference was observed in the discrimination ratio (*F*_5, 47_ = 0.9333, *p* = 0.4680, Figure 3A). Two-way ANOVA revealed a significant treatment effect on exploration time (*F*_5, 86_ = 3.033, *p* = 0.0144, Figure 3B) but no effect of object (*F*_1, 86_ = 2.114, *p* = 0.1496) or interaction (*F*_5, 86_ = 0.5747, *p* = 0.7192) was observed. Further, a significant effect of the treatment (*F*_5, 86_ = 8.705, *p* < 0.0001; Figure 3C) but not the object (*F*_1, 86_ = 1.704, *p* = 0.1948) or interaction (*F*_5, 86_ = 0.5611, *p* = 0.7295) was observed for the number of direct contacts. *Post-hoc* test analysis showed a reduction in the number of direct contacts with a novel object in animals treated with a low dose of mitragynine (Mit 3, *p* = 0.0403). Reduction in the number of direct contacts with a novel object can also be seen in mitragynine- (Mit 3, *p* = 0.0012; Mit 30, *p* = 0.0113) and LKD-treated group (*p* = 0.0113) relative to the morphine-treated group.

### 3.2 Effects of early adolescent kratom exposure on social behaviours

**FIGURE 4:**
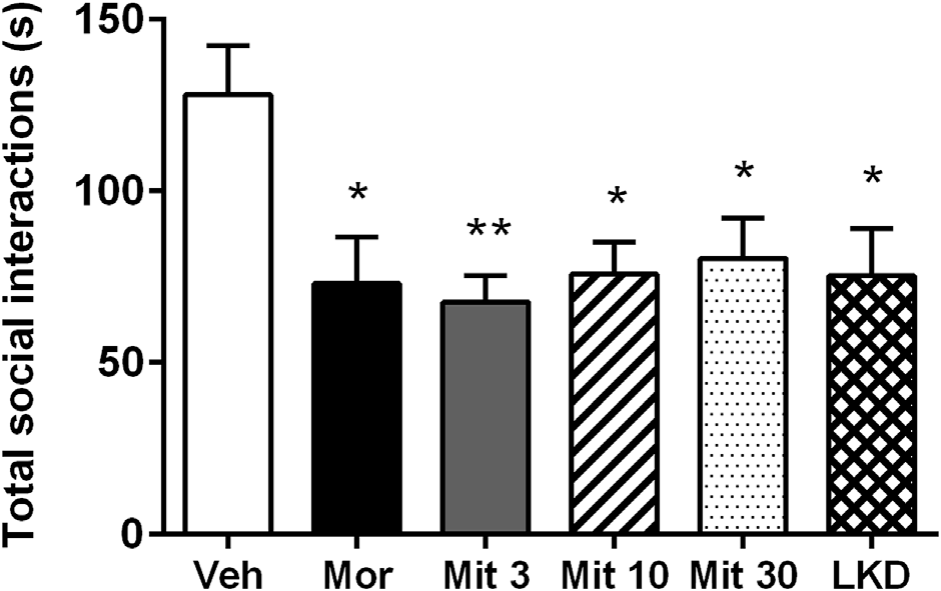
Effects of adolescent kratom exposure on total social interaction. Data are expressed as mean ± SEM (*n* = 8–10). *p < 0.05, **p < 0.01, vs. vehicle-treated group.

One-way ANOVA revealed a significant main effect of treatment on total social interaction time (*F*_5,53_ = 3.882, *p* = 0.0045, Figure 4). Further *post-hoc* analysis showed that morphine (*p* = 0.0189), mitragynine at all doses (Mit 3, *p* = 0.0026; Mit 10, *p* = 0.0117; Mit 30, *p* = 0.0258) and LKD (*p* = 0.0106) significantly reduced the total social interaction time. However, two-way ANOVA revealed no significant effect of treatment (*F*_5, 106_ = 1.999, *p* = 0.0846), type of social interactions (*F*_1, 106_ = 3.346, *p* = 0.0702) and their interaction (*F*_5, 106_ = 1.172, *p* = 0.3281) on the social interaction time as shown in Table 1.

**TABLE 1:** Active and passive social interaction after exposure to kratom during adolescence. Data are expressed as mean ± SEM.

Treatment	Active social interaction (s)	Passive social interaction (s)
Veh	65.74 ± 12.40	62.30 ± 19.92
Mor	44.35 ± 5.88	28.78 ± 15.63
Mit 3	39.39 ± 6.08	28.31 ± 7.51
Mit 10	56.98 ± 10.39	18.87 ± 2.08
Mit 30	52.10 ± 12.97	28.34 ± 11.99
LKD	30.11 ± 8.67	45.21 ± 11.39

### 3.3 Effects of early adolescent kratom exposure on spatial learning and reference memory in Morris water maze task

**FIGURE 5:**
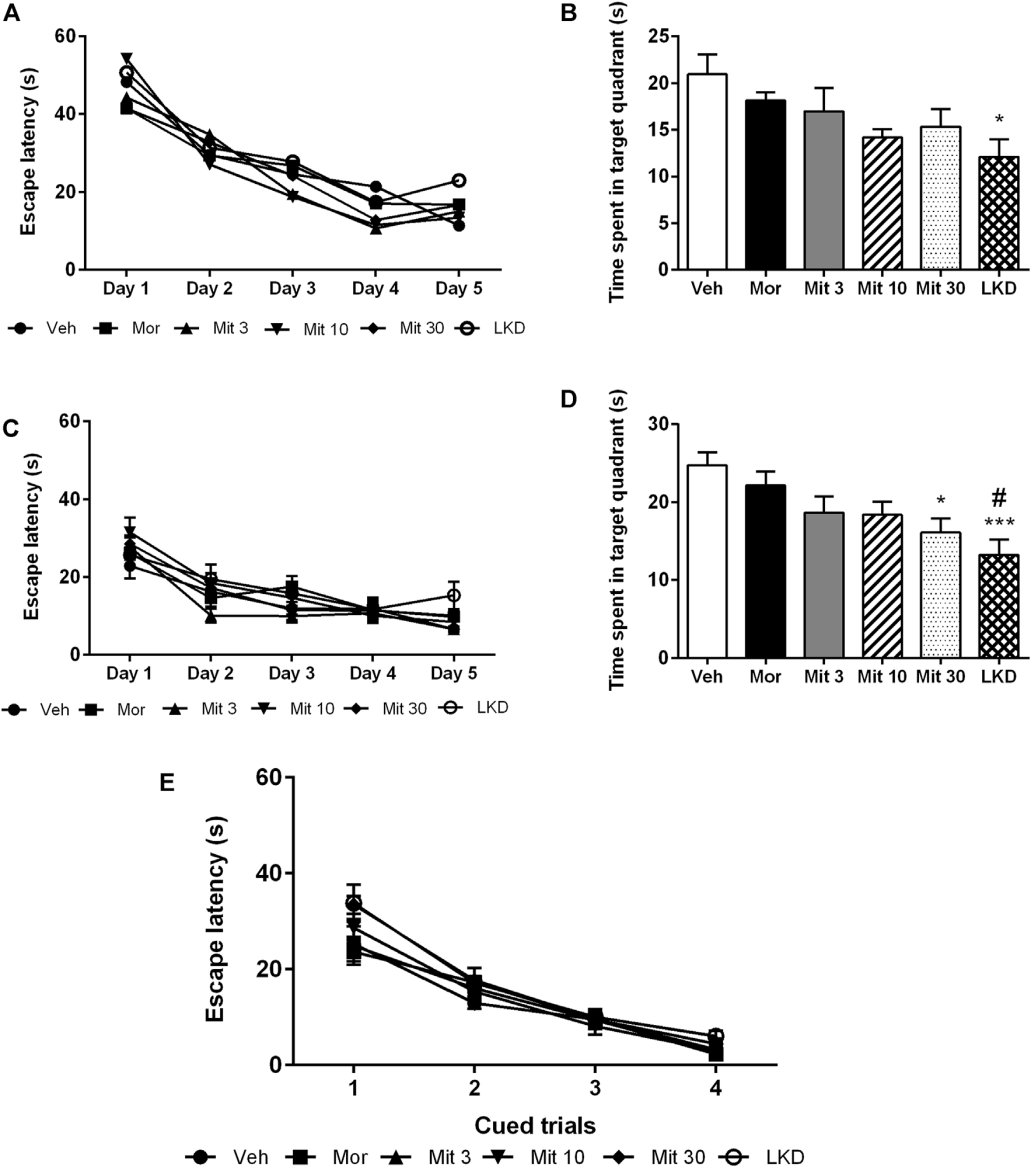
Effects of adolescent kratom exposure on the Morris water maze performance. **(A)** Escape latencies during acquisition trials, **(B)** Time spent in the target quadrant during probe trial, **(C)** Escape latencies during reversal learning, **(D)** Time spent in the target quadrant during reversal probe trial, and **(E)** Escape latencies in the presence of visible platform. Data are expressed as mean ± SEM (*n* = 8–10). *p < 0.05, ***p < 0.001 against vehicle-treated group, #*p* < 0.05 against morphine-treated group.

Analysis of the escape latencies showed no significant effect of treatment (*F*_2.503, 10.01_ = 1.189, *p* = 0.3550, Figure 5A) during acquisition trials which indicates spatial learning was still intact. When the escape platform was removed during the probe trial, time spent in the target quadrant (*F*_5, 45_ = 2.883, *p* = 0.0243, Figure 5B) was affected but only LKD showed a significant reduction (*p* = 0.0252) which suggests a deficit in reference memory. During reversal learning, animals readily learned the new position of the escape platform (*F*_2.503, 10.01_ = 1.189, *p* = 0.3550, Figure 5C). However, the performance of the reversal probe trial was affected (*F*_5, 45_ = 5.119, *p* = 0.0008, Figure 5D) wherein the high dose of mitragynine (Mit30, *p* = 0.0217) and LKD (*p* = 0.0007) significantly reduced the time spent in the target quadrant. Adolescent drug exposure also did not affect general sensorimotor performance, as indicated by the lack of differences in escape latencies when animals were required to swim to a visible platform during cued trials (Figure 5E).

### 3.4 Effects of early adolescent kratom exposure on brain metabolite profiles

#### 3.4.1 Principal component analysis

**FIGURE 6:**
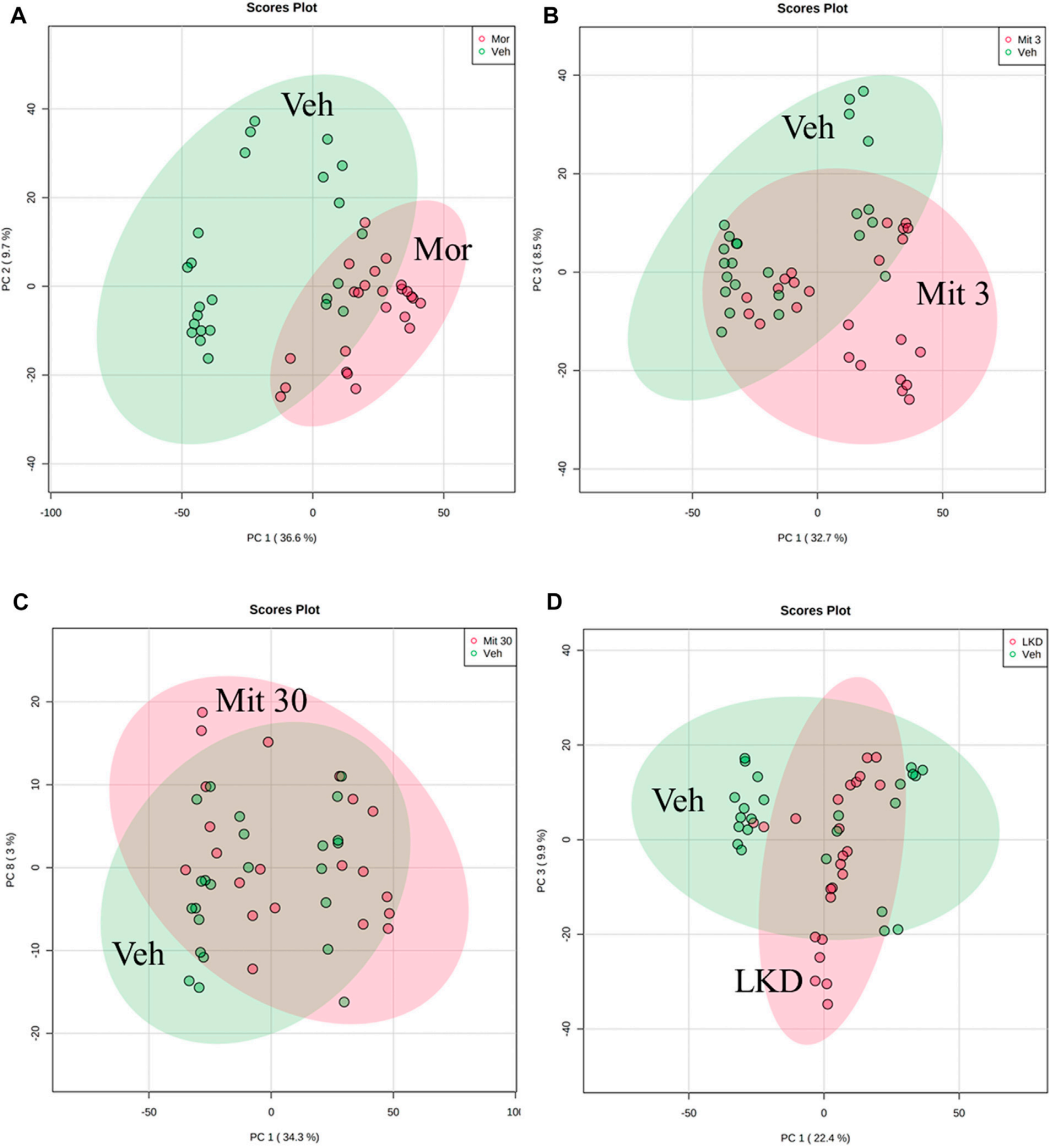
PCA of the metabolite profiles of adolescent rats’ brain samples for the treatment effects of morphine, mitragynine (3 or 30 mg/kg), and LKD versus vehicle. The metabolite profiles for animals treated with **(A)** morphine 5 mg/kg and vehicle show low similarity; **(B)** mitragynine 3 mg/kg and vehicle show low similarity; **(C)** mitragynine 30 mg/kg and vehicle show high similarity; **(D)** LKD and vehicle show low similarity.

The Principal component analysis (PCA) scores plots based on LCMS/QTOF analysis are shown in Figure 6. The metabolite profiles of adolescent rat brain samples represent different isolation between the treatment groups (morphine, mitragynine 3 and 30 mg/kg, and LKD) and the vehicle-treated group. The metabolic profiles of adolescent animals treated with morphine, low dose of mitragynine (3 mg/kg), and LKD were partially clustered with vehicle (Figures 6A,B,D). Meanwhile, animals treated with a high dose of mitragynine (30 mg/kg) showed similar metabolic profiles to the vehicle-treated group (Figure 6C).

#### 3.4.2 Pathways metabolism

**FIGURE 7:**
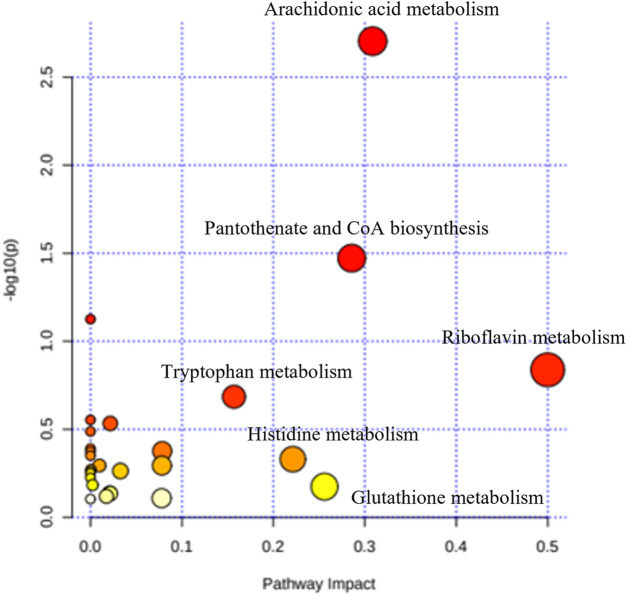
Summary of the pathway analysis of the effects of kratom in adolescent rats’ brain samples.

A total of 306 small molecules were detected from the spectra, significantly different between all five groups. Recursive analysis showed that only 67 metabolites were significant in differential expression after eliminating the false positive data. Metabolites were identified by comparing the mass/charge (m/z) of compounds with METLIN and KEGG databases. The pathways associated with cognitive and behavioural effects of kratom include the metabolisms of arachidonic acid, pantothenate and CoA biosynthesis, and tryptophan with the impact values of 0.31, 0.29, and 0.16, respectively (Figure 7).

**FIGURE 8:**
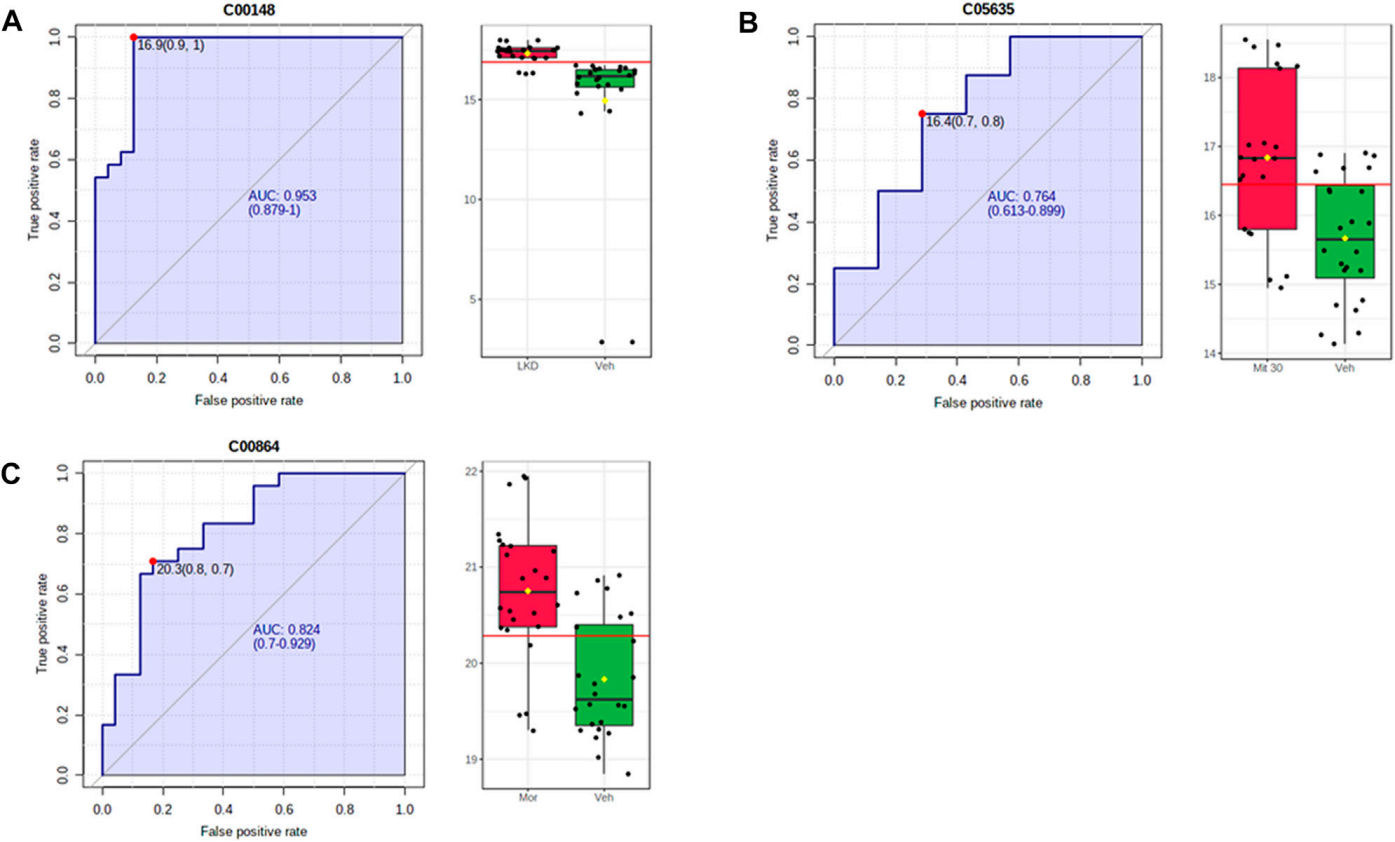
ROC-AUC analysis of the metabolites differentially expressed due to kratom exposure in adolescent rats. The analysis was performed using Metaboanalyst software, by comparing the vehicle- and morphine-treated group, mitragynine 3 mg/kg and 30 mg/kg, and LKD group. Several metabolites were identified to be potential biomarkers with AUC values higher than 0.7. **(A)** L-Proline, **(B)** 5-hydroxyindoleacetic acid, and **(C)** pantothenic acid.

The metabolites such as L-proline, 5-hydroxyindoleacetic acid (5-HIAA), and pantothenic acid with values area under the curve (AUC) of more than 0.7 were categorised as potential biomarkers (Figure 8). The up- and down-regulation of potential biomarkers in their respective pathways are illustrated in Table 2. The metabolite profiles detected in animals treated with mitragynine were similar to the morphine-treated animals, but metabolite profiles of LKD-treated animals were regulated differently, except for 2-Hydroxyhexadecanoic acid.

**TABLE 2:** List of metabolites with significant expression identified in different treatment groups of adolescent rats compared to vehicle-treated group.

Compound	*p*-value	Mor vs. Veh		Mit 3 vs. Veh		Mit 30 vs. Veh		LKD vs. Veh	
		Log FC	Regulation	Log Fc	Regulation	Log FC	Regulation	Log FC	Regulation
L-Proline	3.85E-11	−10.8188	Down	−6.16812	Down	−2.86821	Down	2.608813	Up
5-Hydroxyindoleacetic acid (5-HIAA)	7.93E-09	1.286313	Up	1.554986	Up	1.169034	Up	−0.20167	Down
Pantothenic Acid	2.48E-16	0.918423	Up	0.583046	Up	1.396759	Up	−0.40075	Down
D-Glucosaminide	5.42E-04	−7.46443	Down	−3.67804	Down	−3.86124	Down	0.01435	Up
11(S)- hydroxyeicosatetraenoic acid (HETE)	0.002181	−6.12108	Down	−3.54967	Down	−0.61767	Down	0.163727	Up
2-Hydroxyhexadecanoic acid	0.014457	−7.54603	Down	−0.06709	Down	−1.15408	Down	−4.58898	Down

## 4 Discussion

Here we demonstrated that early exposure (PND31-45) to mitragynine and LKD generally disrupted the cognitive and behavioural performance in adolescent rats with prominent changes in the brain metabolic profile. In a NOR task, mitragynine or LKD did not affect the short-term and long-term recognition memory as seen after 1-h and 24-h retention intervals, respectively. However, mitragynine caused a reduction in the number of direct contacts with both familiar and novel objects. It is possible that repeated mitragynine exposure alters the neurobiological mechanism(s) related to anxiety and stress (Farah et al., 2011; Johnson et al., 2020) resulting in less motivation to explore the objects, yet this notion needs to be further investigated. Furthermore, the NOR task is a sensitive behavioural paradigm capable of detecting subtle differences in memory (discrimination) and exploratory performance. As a result, it is susceptible to potential biases such as stress or drug side effects (Akkerman et al., 2012).

The social interaction test has been used extensively for assessing social behaviour in rodents, wherein the reduction in social interaction will reflect the increase in the emotional state that leads to dysfunctional social reward (Brancato et al., 2021). In the present study, we found that mitragynine at all doses and LKD cause a decrease in the total social interaction indicating social withdrawal after the drug exposure (Almeida et al., 2013). However, no difference was observed in the active and passive interactions between groups. Although the previous finding reported that acute mitragynine may have anxiolytic effects (Farah et al., 2011; Hazim et al., 2014; Yusoff et al., 2016), our present data showed a lack of social interaction which suggests that mitragynine, regardless of the doses and LKD may induce anxiogenic effects in adolescent rats, affecting social behaviour in a novel environment. A growing literature investigating the effects of drug abuse on social behaviour in rodents demonstrates that drugs influence social behaviour (Ayranci et al., 2014; Becker et al., 2017; Listos et al., 2022), especially in adolescence (Kuhn, 2015). For instance, repeated alcohol exposure increases anxiety-like behaviour under social circumstances in adolescents but not in adult animals, as indicated by a decrease in social preference (Varlinskaya and Spear, 2007).

Our findings further showed that adolescent exposure to mitragynine and LKD did not impair spatial learning. Still, LKD caused a deficit in reference memory in MWM as evidenced by the decrease in the time spent in the target quadrant during the probe trial. When the escape platform was repositioned in the reversal MWM, new spatial learning was not affected, with the slope of escape latencies becoming less steep indicating that the animal readily learned during the acquisition trials. However, a high dose of mitragynine and LKD caused a deficit in the new reference memory. This finding indicates that spatial working memory during acquisition learning may not be a crucial step toward forming a reference memory (Niewoehner et al., 2007; Bannerman et al., 2008), which may explain the distinct performance in acquisition and probe trials of MWM in the present study. This finding also supports the notion that spatial learning and reference memory formation are subserved by different neural mechanisms (Izquierdo et al., 2000).

Since mitragynine has been classified as an atypical opioid agonist (Kruegel et al., 2016; Obeng et al., 2020), we used morphine as a comparison as it may elicit similar pharmacological effects. Furthermore, several preclinical studies have shown that morphine treatment can induce social and memory deficits in animal models (Rabbani et al., 2009; Naghibi et al., 2012; Ellis et al., 2020; Becker et al., 2021). However, our behavioural findings showed that morphine caused a marked deficit in social behaviour which is consistent with earlier studies (Goeldner et al., 2011; Ayranci et al., 2014; Becker et al., 2021), but did not cause any significant changes in recognition memory, spatial learning, and reference memory. This may be attributed to the difference in morphine exposure paradigms such as dosage, duration of drug exposure (Wang et al., 2017), and length of withdrawal (Becker et al., 2021). Furthermore, it can be suggested that different cognitive domains may have different susceptibility to the impairing effects of morphine.

Previously, extensive studies on kratom-induced cognitive and behavioural changes have been performed in adult animals. Mitragynine has been shown to impair the working memory of mice in object placement task (Apryani et al., 2010) and different stages of learning and memory in inhibitory avoidance task (Yusoff et al., 2016). In the MWM task, mitragynine was shown to impair spatial learning but not reference memory (Hassan et al., 2019). A recent study also showed that chronic escalating high doses of mitragynine led to the impairment of place learning and its reversal using the IntelliCage^®^ system (Iman et al., 2021). Electrophysiological study also indicated that mitragynine can cause a disruption in hippocampal synaptic transmission and inhibition of LTP induction (Hassan et al., 2019). However, these findings are unlikely to represent the effects of kratom in adolescents since their brain is still developing and neurodevelopment may be compromised.

Early adolescent exposure to substance abuse can interfere with ongoing neurodevelopment that may induce changes in the neurobiological system and subsequent cognitive and behavioural performance (Schwarz and Bilbo, 2013; Fernandez and Savage, 2017; Jordan and Andersen, 2017). Using metabolomics, the neurochemical disruptions associated with substance abuse can be elucidated as the metabolome reflects the various neurochemical processes that occur within a biological system (Caspani et al., 2021). Thus, in the present study, we profiled the brain metabolomes of the kratom-treated adolescent animals. Pathway analysis revealed several altered metabolic pathways which were differentially expressed in the brain after mitragynine and LKD exposure. These include the pathways involved in the metabolism of arachidonic acid, pantothenate and coenzyme A, and tryptophan.

Drug use may render the brain more vulnerable to inflammation and subsequent neuropathology. There is considerable interest in how drug use interacts with inflammatory processes, leading to brain dysfunction, cognitive control impairment, and eventually promoting drug-use behaviour (Keen and Turner, 2014; Kohno et al., 2019). One of the mechanisms is the arachidonic acid pathway which plays a crucial role in many inflammatory diseases, cardiovascular and cancer biology (Borin et al., 2017; Wang et al., 2021; Zhou et al., 2021). Arachidonic acid can be metabolized by three different enzyme systems, i.e., cyclooxygenases (COXs), lipoxygenases (LOXs), and cytochrome P450 (CYP) enzymes to produce a diverse range of biologically active fatty acid mediators (Wang et al., 2021). Drug-induced changes in the arachidonic acid metabolism are also associated with neuroplasticity and signal transduction (Colbert and Pan, 1999; Rao et al., 2013; Sambra et al., 2021), wherein an increase in arachidonic acid signaling has been linked to cognitive impairment (Rao et al., 2013). Experimental excitotoxicity and neuroinflammation also selectively increase brain arachidonic acid metabolism (Rapoport, 2008). According to a prior study, persistent D-amphetamine use and its withdrawal reduce brain arachidonic acid consumption and signaling due to neuroplastic alterations that may correlate to depressive behaviours after drug withdrawal (Bhattacharjee et al., 2008). Gopaldas et al. (2019) also found that arachidonic acid status may influence depression pathophysiology *via* effects on serotonin transport. Therefore, the dysregulation of the arachidonic acid pathway may partly explain the general cognitive deficit and lack of social interaction in the present study. This dysregulation may also indicate that the neurodevelopment in adolescents exposed to kratom has been compromised as their metabolite is involved in various brain processes, including synaptic growth and transmission, gene expression, membrane fluidity and flexibility, apoptosis, neuroinflammation, and excitotoxicity (Duplus and Forest, 2002; Ryan et al., 2014; Tallima and El Ridi, 2018).

Pantothenic acid is required for the synthesis of CoA, which plays a role in many major metabolic pathways, including carboxylic and fatty acid metabolism (Czumaj et al., 2020). However, an increase in the pantothenate-CoA activity may lead to inappropriate consumption of biological defence resources such as pro-inflammatory cytokines, adhesion molecules, and acute response proteins related to inflammation (Lee et al., 2018). Reduced pantothenic acid levels may result in insufficient CoA production for the tricarboxylic acid (TCA) cycle to function properly (Scholefield et al., 2021). Aside from its role in oxidative metabolism, CoA helps to maintain the structure and function of brain cells by participating in the synthesis of cholesterol, amino acids, phospholipids, and fatty acids (Ismail et al., 2020). Notably, pantothenic acid is also involved in the synthesis of multiple neurotransmitters and steroid hormones *via* CoA-dependent pathways (Lee et al., 2018), and pantothenic acid’s brain level was reduced in neurodegenerative diseases like Alzheimer’s, Huntington’s, and Parkinson’s, in which cognitive performance was impaired (Scholefield et al., 2021). Hence, it is very likely that the changes in the pantothenate and CoA regulation may impact the cognitive and behavioural performance of adolescents exposed to mitragynine or LKD.

The tryptophan metabolism is balanced by two distinct enzymes: tryptophan hydroxylase and indoleamine 2, 3-dioxygenase (IDO), which act in the serotonin and kynurenine pathways, respectively (Jayamohanan et al., 2019; Connell et al., 2022). A shift in tryptophan metabolism to the kynurenine pathway reduces the availability of tryptophan in the serotonin pathway and subsequently affects the serotonin level which can be confirmed by detecting its final metabolite, 5-hydroxyindoleacetic acid (5-HIAA, Connell et al., 2022; Jayamohanan et al., 2019). In the present study, 5-HIAA was upregulated in animals treated with morphine and mitragynine but downregulated in animals treated with LKD. The serotonin system is known to play a crucial role in modulating the physiology and behaviour in health and disease states (Ren et al., 2018; Konova and Kohout, 2021). Low serotonin is closely associated with declines in learning and memory and other neurological disorders such as depression (Cowen and Sherwood, 2013). In rodents, oral administration of tryptophan led to an increase in serotonin neurotransmission with an improvement in memory acquisition, consolidation, and retrieval, whilst daily tryptophan injections improved spatial memory (Haider et al., 2007). Hence, it can be speculated that the imbalance of tryptophan metabolism may underlie the cognitive and behavioural decline *via* the serotonin system following exposure to the mitragynine and LKD in the present study.

Aside from 5-HIAA and pantothenic acid, we also identified the amino acid L-proline as one of the potential biomarkers, with AUC values higher than 0.7. Proline can be found in abundance in the central nervous system, with proline transporters highly expressed within synaptic terminals of a subset of glutamatergic neurons in the brain (Zipp et al., 2014), indicating its pivotal role in the regulation of synaptic transmission. Elevated proline levels affect the glutamatergic transmission, depolarize neurons, increase synaptic activity, alter cognitive tasks, sensorimotor gating, and locomotor activity in animal models (Martin et al., 1992; Phang, 2019; Jones et al., 2021). In humans, a condition characterised by abnormally elevated proline levels called hyperprolinemia has been associated with epilepsy, seizures, and cognitive decline. Even though various studies demonstrate the key roles of proline in brain function and neurological disorders, the role of proline metabolism during drug exposure remains largely unknown (Jones et al., 2021) and merits further investigation.

Generally, adolescent animals treated with morphine, mitragynine, and LKD exhibit a similar pattern of cognitive and behavioural outcomes. However, metabolomic data reveals that mitragynine and morphine had a similar expression pattern of the potential biomarkers, but not the LKD. Therefore, it seems likely that the various constituents within LKD such as mitragynine, speciociliatine, paynanthine, and others (Wilson et al., 2020) may act synergistically in producing pharmacological effects that contradict its single compound like mitragynine in the biological system (Zhou et al., 2016). Receptor binding studies have also shown that individual compounds in kratom may have different binding affinities and efficacies to various sub-type receptors of the opioid, adrenergic and serotonergic receptors (Kruegel et al., 2016; Obeng et al., 2020, 2021; Leon et al., 2021). The conversion of mitragynine to a more potent mu-opioid receptor agonist, 7-hydroxymitragynine, and a multifunctional mu agonist/delta-kappa antagonist, mitragynine pseudoindoxyl *via* a CYP3A-mediated pathway, and a potentially toxic metabolite, 3-dehydromitragynine, *via* a non-CYP oxidation pathway may also implicate the pharmacological effects *in vivo* (Chakraborty et al., 2021; Hill et al., 2022).

Another factor that may implicate our findings is the timing for the behavioural testing and brain extractions for metabolomic analysis. In the present study, behavioural testing was conducted 24 h after the drug cessation and continued until day 15, which may coincide with the molecular neuroadaptations. Previous studies have demonstrated that acute and protracted drug withdrawal would result in different behavioural and molecular outcomes (Becker et al., 2017; Listos et al., 2022). Hence, it is possible that the state of dependence, addiction, or withdrawal of a particular drug may yield different behavioural and metabolomic outcomes.

The present study uses the whole brain tissue homogenate for the metabolomic analysis which only detects changes in the overall metabolite pool. Thus, the highly localized effects will not be detectable in the tissue homogenate as it is impossible to differentiate, for instance, the metabolic changes in specific cognition-associated brain regions. However, the significant changes in the metabolite profile observed here clearly show the method’s value as a screening tool. Furthermore, these findings highlight the importance of additional metabolomic studies in understanding the roles of the implicated metabolites following kratom exposure in adolescents.

In summary, adolescent kratom exposure particularly mitragynine and LKD may cause selective cognitive and behavioural deficits. The brain metabolite profiles further suggest that the altered metabolic pathway (i.e., arachidonic acid, pantothenate and CoA, and tryptophan) may underlie the kratom-induced cognitive and behavioural deficits. Together, these findings demonstrate that adolescents’ brain is sensitive to the impact of early kratom exposure during this critical development period.

## Data Availability

The original contributions presented in the study are included in the article/Supplementary Material, further inquiries can be directed to the corresponding author.
