# Supplementary material for: Adolescent kratom exposure affects cognitive behaviours and brain metabolite profiles in Sprague-Dawley rats
Source: Front Pharmacol. 2022 Nov 28;13:1057423. doi: 10.3389/fphar.2022.1057423 (PMC9744228; doi:10.3389/fphar.2022.1057423)
Supplement: Supplementary file 1 [file DataSheet1.docx]

**Supp. Material 1**

**1.0 Plant material**

Approximately 1 kg of fresh leaves of kratom (*Mitragyna speciosa* Korth.) were collected from a local plantation at Permatang Rawa, Penang, Malaysia. A voucher specimen [NEL-(K2)-2019(O2)] was deposited at the herbarium of the School of Biological Sciences, Universiti Sains Malaysia.

**2.0 Preparation of kratom decoction**

Fresh kratom leaves (1 kg) were harvested and rinsed with tap water before being cut into tiny pieces and placed in a boiling pot of water (12 L). The leaves were brewed for about 2 hours at a constant heat until the water was reduced to about one-third of their original volume. The solution was then allowed to cool before being filtered and freeze-dried to produce a lyophilised kratom decoction (LKD) extract. The LKD extract was maintained at -80°C before use.

**3.0 HPLC profiling**

**3.1 Chemicals and Reagents**

Mitragynine (purity > 98%) was extracted from kratom leaves according to our in-house method (Chear et al., 2021; Domnic et al., 2021). Solvents—acetonitrile and methanol used for HPLC analysis were of HPLC grade (Merck, Germany). Formic acid (98–100%) was purchased from Merck (Germany). Deionized water (18.2 MΩ) was used for the HPLC analysis.

**3.2 Analytical Method**

The mitragynine content in the prepared LKD sample was determined using a validated HPLC method as described in our previous study (Saref et al., 2019; Damodaran et al., 2021). In brief, the HPLC analysis was performed on an Agilent 1200 series HPLC system coupled with a photodiode array detector (Agilent, CA, USA). A stock solution of LKD extract (1000 µg/mL) was prepared in methanol, centrifuged, and filtered to remove undissolved particles. The separation of the extract was conducted on an Inertsil C8-3 column (4.6 x 150 mm, 5 µm) (GL Sciences Inc. Japan) with a total run time of 27 mins. The mobile phase was a mixture of 0.1% formic acid (A) and acetonitrile (B) running at a gradient method: 0-2 mins – 80% (A) and 20% (B), 2.1-7.0 mins – 70% (A) and 30% (B), 7.1-12.0 mins – 50% (A) and 50% (B), 12.1 to 20.0 mins – 100% (B), 20.1 to 27 mins – 80% (A) and 20% (B) with a flow rate of 1 mL/min. The injection volume for extract and standard was 10 µL. Mitragynine was detected using an Agilent photodiode detector at λ_max_ 250 nm. The targeted peak (mitragynine) in the HPLC chromatogram was identified by comparing the retention time and UV spectrum with the reference standard. A linear calibration curve of mitragynine standard was prepared from 3.125 to 100 µg/mL. Data analysis was done by using the ChemStation LC3D software.

**4.0 Result**

**Mitragynine content**

The HPLC profile for LKD and the detected mitragynine is given in **Figure 1A**. The chromatographic peak of mitragynine in the extract was identified by matching its retention time with the reference standard. The UV profile of mitragynine standard and their corresponded peaks in the LKD are provided in **Figure 1B**. Based on HPLC analysis, mitragynine was detected at a retention time, 8.40 min. A linear calibration curve of mitragynine standard was then plotted from 3.125 to 100 µg/mL, with a correlation coefficient value, R^2^ > 0.9999, and an equation, y = 15.979× + 7.1522.

In this study, 4000 mL of concentrated kratom decoction (extracted from 1kg kratom leaves) was lyophilised to yield 61.71 g extract. Subsequently, the amount of mitragynine detected in the LKD (1000 µg/mL) was 20.22 ± 2.30 μg/mL. According to this quantitative analysis of LKD for mitragynine content (0.02022 mg/mg), 1.484 g/kg of LKD would be required to achieve the equivalent dose of mitragynine (30 mg/kg).

**B**

**A**

Figure 1A: The HPLC chromatogram of (A): a lyophilised kratom decoction; (B) a mitragynine standard detected at λ_max_ 250 nm.

**B**

**A**

Figure 1B: The UV profile of (**A**) mitragynine peak in the lyophilised decoction; (**B**) mitragynine standard detected at λ_max_ 250 nm.
